# From Biomarkers to Precision Medicine in Neurodegenerative Diseases: Where Are We?

**DOI:** 10.3390/jcm11154515

**Published:** 2022-08-03

**Authors:** Giacomo Tondo, Fabiola De Marchi

**Affiliations:** 1 Neurology Unit, S. Andrea Hospital, Department of Translational Medicine, University of Piemonte Orientale, 13100 Vercelli, Italy; giacomo.tondo85@gmail.com; 2 Division of Neuroscience, IRCCS San Raffaele Scientific Institute, 20132 Milan, Italy; 3 ALS Centre, Neurology Unit, Maggiore della Carità Hospital, Department of Translational Medicine, University of Piemonte Orientale, 28100 Novara, Italy

The biomarkers era grew in the last two decades when several technical and methodological advances have improved the research in neurodegenerative diseases. All neurodegenerative conditions are progressive and debilitating disorders due to premature and irreversible neuronal loss, leading to consequent clinical deficits. The improvement in life expectancy entails a worldwide increase in these disorders, aging being the greatest risk factor for these incurable conditions. Identifying early and reliable markers for neurodegenerative diseases potentially allows the clinician to reach a timely diagnosis, provide disease staging and monitoring disease progression, increase prognostic precision, and reduce the health system burden. All these goals are essential to design clinical trials and test novel therapeutic strategies correctly.

Most neurodegenerative diseases share similar pathogenic events, starting from the pathological deposition of misfolded proteins and leading to progressive neuronal damage associated with altered neuroinflammatory response. The clarification of the common pathogenic processes may open the development of effective therapeutic approaches in a field still lacking disease-modifying drugs. The current therapeutic strategies rely only on symptomatic treatment, which can stabilize or temporarily slow the clinical worsening, but they do not impact the underlying disease mechanism. For these reasons, searching for disease-specific biomarkers is a crucial step in this direction.

Currently, most biomarkers are classified based on the pathogenic mechanism they can underline. Biomarkers of pathology, including markers for amyloid-β (Aβ) pathology and tau pathology, are primarily used in Alzheimer’s disease (AD) and the Frontotemporal lobar degeneration (FTLD) spectrum. They can be addressed mainly through cerebrospinal fluid (CSF), blood analysis, and positron emission tomography (PET) imaging. For Parkinson’s disease (PD) and parkinsonism, to date, the search for α-synuclein can be feasible in CSF, while no PET ligands selective for α-synuclein aggregates have been validated in humans [1]. In addition to pathology markers, not-specific neurodegeneration markers can play an important role in neurodegenerative disorders. Neurofilament light chain (NfL), a neuronal cytoplasmic protein highly expressed in large caliber myelinated axons, is a promising marker for axonal degeneration, potentially measurable both in CSF and blood. Neuroinflammation, a common pathogenetic mechanism linked with neurodegeneration, leads to alterations in the relationship between neurons and glial cells. Although the protective or harmful role of neuroinflammation in neurodegenerative diseases is not yet fully understood, it seems sure that the activation of microglia and astrocytes is a key event in the neurodegenerative cascade [2].

Alzheimer’s disease (AD) is the most common neurodegenerative disease, and the biomarkers research has been primarily focused on this condition. The ATN system, based on the in vivo evidence of Aβ (A), tau pathology (T), and neurodegenerative changes (N), revolutionized the concept of the clinical diagnosis shifting to a biological definition of the AD spectrum. The AD continuum ranges from the overt dementia phase to the preclinical, asymptomatic, or subtle symptomatic phase associated with AD neuropathological changes. A precise diagnostic process along the AD spectrum is essential to provide optimal management for the patients, avoiding misdiagnosis and unsuccessful treatment [3].

The diagnostic role of CSF biomarkers has been largely validated for dementia disorders. CSF biomarkers including phosphorylated-tau, total-tau, Aβ-42, Aβ-40, and NfL may support the in vivo identification of AD and FTLD neuropathology and neurodegenerative-related changes, facilitating differential diagnosis [4]. In the study of Mattsson-Carlgren and colleagues, the CSF levels of Aβ, tau, and NfL were correlated with autopsy-confirmed neuropathological changes in a cohort including AD and FTLD patients. The ratio between phosphorylated-tau and Aβ-42 showed the highest overall diagnostic performance, with an area under the curve of 0.95–0.96. In addition, distinct biomarker patterns emerged in the different FTLD subtypes: for example, increased NfL and a reduced ratio between phosphorylated-tau and total-tau were observed in the FTLD–TAR DNA-binding protein 43 subtype, while higher reduction of total-tau was observed in progressive supranuclear palsy compared with other FTLD variants [5].

Recent studies on people at risk for developing dementia pointed out this population’s heterogeneity, which can be composed of different underlying etiologies but mainly include people without signs of neurodegeneration, as detected by FDG-PET [6]. The search for biomarkers in clinical practice in asymptomatic or subtle symptomatic individuals is heatedly debated since the potential identification of people at risk for untreatable neurodegenerative conditions implies considerable ethical concerns. Nevertheless, longitudinal findings demonstrated a higher risk of progressing to dementia and developing a steeper cognitive decline for cognitively unimpaired individuals along the AD continuum than individuals with normal biomarkers [7]. In research, identifying subjects showing disease-specific neuropathological alterations without overt clinical impairment represents the best opportunity to act on the disease course at the beginning of neurodegenerative changes. This aim would be achievable through a mass screening using easily accessible blood tests able to provide meaningful information about the pathophysiological basis of neurodegeneration. However, validation on large cohorts to confirm the reliability of the test is still warranted, and this is only an experimental approach. The study of Pereira and colleagues investigated blood markers of neurodegeneration and pathology in a large cohort of cognitively unimpaired AD dementia and non-AD dementia patients from the Swedish BioFINDER-2 study, combining plasma Aβ, tau, and NfL measurements to longitudinal imaging and cognitive data. The first result was the evidence that a reduced baseline Aβ42/Aβ40 plasma level independently predicted the longitudinal increase in cerebral amyloid load as detected by amyloid-PET. Similarly, increased plasma phosphorylated-tau217 levels predicted longitudinal tau accumulation as detected by tau-PET and cerebral brain atrophy. In addition, plasma phosphorylated-tau217 levels predicted cognitive decline. Furthermore, these two plasma markers were highly correlated with the CSF biomarkers. Interestingly, no association was found between plasma marker levels and longitudinal brain changes in amyloid-negative patients with a non-Alzheimer’s neurodegenerative disorder, suggesting a possible high specificity of plasma Aβ42/Aβ40 and phosphorylated-tau217 for AD [8].

NfLs represent intriguing candidates in the prognostic workup of neurodegenerative diseases. While the turnover of NfL in healthy aging brains is slow, it is increased, in different disease-related behaviors, in several neurodegenerative conditions. To date, NfLs are largely used in Multiple Sclerosis, various dementia conditions such as AD and FTLD, and several other neurodegenerative diseases, including Amyotrophic Lateral Sclerosis (ALS). A study enrolling 231 patients with suspected ALS showed high diagnostic performance in discriminating patients from mimics (significantly higher plasma and CSF NfL values compared with ALS mimics), although CSF NfL performed slightly better than plasma NfL. In addition, higher baseline NfL levels in CSF and plasma were demonstrated in ALS patients with shorter survival. However, longitudinal plasmatic analyses of NfL showed stable levels of NfL over the disease course, questioning the role of this marker in staging the disease. This last finding can be influenced by several factors and needs further longitudinal research, but its eventual reduction in clinical trials would be a significant slowdown in the neurodegenerative process [9].

Despite not-univocal results, the longitudinal changes in biomarker measurements may help in staging the disease progression and eventually in monitoring response to therapy. Neuroinflammation is now recognized as a critical element in the neurodegenerative process. Microglia activation can be visualized in vivo by PET imaging of neuroinflammation, specifically using tracers for the 18 kDa translocator protein (TSPO), overexpressed by activated glial cells. TSPO-PET potentially allows for visualization changes in neuroinflammatory responses over the disease course, thus revealing different phases of the neurodegenerative process. The study of microglia activation has provided intriguing results in Mild Cognitive Impairment (MCI), a transitional condition between normal aging and dementia. MCI individuals showed higher baseline TSPO-PET signals than controls, with a longitudinal decrease of microglia activation, despite an increased cerebral amyloid burden detected by amyloid-PET. In addition, AD patients showed higher baseline TSPO-PET signal than controls, but with a further increase over time. These findings supported the hypothesis of a double peak in microglia activation along the AD trajectory, with an initial, protective activation in MCI subjects, possibly contrasting neurodegenerative changes, and a late, proinflammatory activation in AD patients with deleterious consequences on neuronal functions [10]. TSPO-PET can be a unique tool to detect microglia activation in other brain disorders than AD and even in pre-symptomatic or asymptomatic individuals carrying a pathogenic mutation for neurodegenerative diseases. Mutations in the SOD1 gene are one of the main causes of familial ALS and are associated, at the molecular level, with increased oxidative stress and inflammation. Microglia activation has been studied in asymptomatic and symptomatic SOD1 mutated carriers, resulting in an increased TSPO-PET signal in both groups compared with controls. These results suggested that microglia activation may be associated with neurodegeneration already in the asymptomatic phase of the process [11].

As shown, identifying markers able to stage the disease or monitor disease progression may be crucial in clinical trials. Identifying possible clinical trajectories along the neurodegenerative process offers the possibility of testing therapeutic strategies in a pre-symptomatic phase. For several years, many clinical trials failed; however, it is not certain whether these failed due to the actual ineffectiveness of the trial or difficulty in identifying any clinical or biological variations due to the absence of specific disease markers. Likewise, in recent clinical trial designs (e.g., in the VALOR study to evaluate the role of Tofersen in ALS-SOD1 patients, NCT02623699), biomarkers have been included among primary and secondary outcomes, highlighting the importance of biomarkers incorporation in the early phase of drug development.

However, some considerations about the best biomarkers, or combination of biomarkers, to include in clinical trials are needed. Firstly, the selected biomarkers must be easily measurable and reproducible in several centers, considering the growing spread of neurodegenerative diseases. In this regard, NfL can represent a favorable biomarker for the limited costs and the recent simple technique to dose them both in CSF and plasma [12]. Secondly, the ideal biomarker should correlate with some clinical parameters and must serve as an index of clinical response to have the great opportunity to monitor therapy response in the clinical trials and to predict prognosis. We should mention, as an actually negative example, the failure of the aducanumab trial, where the results obtained by the PET imaging did not correlate to a clinical benefit.

In recent decades researchers have been trying to enhance the development of reliable biomarkers for neurodegenerative diseases, albeit with conflicting and not consistently optimal results, while the medical practice has been moving toward precision medicine. However, there is still an urgent need to integrate the disease-specific biomarkers in clinical practice and develop effective disease-modifying therapies. In addition, the current identification of possible clinical trajectories along the neurodegenerative process in several subjects, such as patients with subjective cognitive complaints or patients who carried ALS mutations, offers the possibility of testing therapeutic strategies in a pre-symptomatic phase. Similarly, the investigation of neuroinflammatory responses represents a possibly invaluable tool in monitoring disease progression and predicting clinical outcomes.

We are proposing a research topic focused on biomarkers in neurodegenerative diseases, aiming to gather evidence on the molecular mechanisms involved in neurodegeneration. This Special Issue represents a chance for clinicians and researchers to provide new insights into the pathogenesis of neurodegenerative diseases, encouraging the development of research for novel potential therapeutic interventions and patient management.
